# Necrology

**Published:** 1900-06

**Authors:** 


### NECROLOGY.

At Philadelphia, May 12, 1900, Dr. A. Tegtmier, aged seventy-three years. For more than thirty years he had been a practitioner of veterinary medicine, though a non-graduate, employing homœopathic measures in his practice.

On May 6th, at Winsted, Conn., Dr. E. M. Heath, graduate of the Chicago Veterinary College, class of 1897. Dr. Heath was also a member of the American and Connecticut Veterinary Associations ; was in attendance at the last meeting of the American Veterinary Medical Association in New York, and since that time has rapidly declined in health from tuberculosis, leaving a wife and two small children. Current report indicates the belief that Dr. Heath contracted tuberculosis from his attendance upon examination and treatment of cattle.

---

Dr. J. H. McNeal, of the Bureau of Animal Industry, has been transferred from Boston to Buffalo, N. Y. It would seem that his stay among the bean-eaters was not as congenial to his taste as his sojourn among the Buffalonians.

Dr. F. C. Grenside, of New York City, is among the contributors to a fund for a mounted police medal and indorsement. It is hoped that the fund will ultimately provide for a free bed in one of New York's city hospitals for anyone injured in the performance of duty.

Dr. H. D. Gill, of New York City, takes an active part in matinée road races.

